# Multi-Camera Vehicle Tracking Using Edge Computing and Low-Power Communication

**DOI:** 10.3390/s20113334

**Published:** 2020-06-11

**Authors:** Maciej Nikodem, Mariusz Słabicki, Tomasz Surmacz, Paweł Mrówka, Cezary Dołęga

**Affiliations:** 1 Faculty of Electronics, Wrocław University of Science and Technology, Wyb.Wyspiańskiego 27, 50-370 Wrocław, Poland; tomasz.surmacz@pwr.edu.pl; 2 Institute of Theoretical and Applied Informatics, Polish Academy of Sciences, ul. Bałtycka 5, 44-100 Gliwice, Poland; mslabicki@iitis.pl; 3 Neurosoft Sp. z o.o., ul. Życzliwa 8, 53-030 Wrocław, Poland; pawel.mrowka@neurosoft.pl (P.M.); cezary.dolega@neurosoft.pl (C.D.)

**Keywords:** vehicle detection, vehicle tracking, multi-target multi-camera tracking, edge processing, IoT, low-power short-range radio

## Abstract

Typical approaches to visual vehicle tracking across large area require several cameras and complex algorithms to detect, identify and track the vehicle route. Due to memory requirements, computational complexity and hardware constrains, the video images are transmitted to a dedicated workstation equipped with powerful graphic processing units. However, this requires large volumes of data to be transmitted and may raise privacy issues. This paper presents a dedicated deep learning detection and tracking algorithms that can be run directly on the camera’s embedded system. This method significantly reduces the stream of data from the cameras, reduces the required communication bandwidth and expands the range of communication technologies to use. Consequently, it allows to use short-range radio communication to transmit vehicle-related information directly between the cameras, and implement the multi-camera tracking directly in the cameras. The proposed solution includes detection and tracking algorithms, and a dedicated low-power short-range communication for multi-target multi-camera tracking systems that can be applied in parking and intersection scenarios. System components were evaluated in various scenarios including different environmental and weather conditions.

## 1. Introduction

Traffic monitoring is an emerging topic, especially as it may be a crucial part of smart-city concept. Volume of car traffic combined with ubiquitous wireless communication and low-priced electronic devices create a new range of applications which belong to the scope of traffic monitoring [1]. Those applications include vehicle detection and classification, detection of ADR plates, parking spot occupancy detection, traffic rules enforcement, and many others. Depending on the application, the system have different Quality of Service requirements. As some of those applications are responsible for traffic management, they need to operate in efficient and reliable manner in various conditions [2].

Over the years a number of technologies and systems were proposed for vehicle traffic monitoring [3,4,5,6]. For example, inductive loops or sonar/microwave-based detectors are popular at parking barriers, traffic lights and highways. However, their applications are limited, as they are less universal compared to video monitoring. The most important advantages of video-based systems are: relatively low installation cost, lack of need for complex construction work during installation, and ability to perform wide spectrum of tasks (e.g., detection, classification, tracking, traffic law violations monitoring) in various environments and weather conditions [4]. Thanks to its easy installation, video based systems may be considered for both short and long-term deployments. In the latter case, low-energy consumption is an important factor since in many applications providing external power may be infeasible and the cameras need to operate on batteries. Temporary traffic counting is an example of such short-term application, where battery operation capability is a benefit. Another interesting use-case for video based systems is traffic monitoring over a car parking or an intersection. In these scenarios, vehicles enter and leave the monitored area by one or more roads, and usually more than one camera is required due to the size of the monitored area.

One of the challenges in implementing camera-based traffic monitoring systems is their computational complexity. Sophisticated artificial intelligence algorithms, which often process high-resolution images from cameras in real-time, have high resource usage. Due to this fact, they cannot be implemented in constrained embedded systems or even in single-board computers. Naturally, the usage of images with lower resolution minimizes resources required for image processing algorithms, but affects the image quality and hinders some of the applications, including automatic number plate recognition (ANPR). One way to address this issue is to adjust the algorithms or modify approach (e.g., instead of ANPR, unique car features may be used for vehicle tracking [7,8]) but this often leads to increased computational complexity. Another approach is to implement methods that detect vehicles based on detecting differences in subsequent frames of the video [9]. This allows to decrease complexity but requires fine-tuning for each deployment and is sensitive to changes in the camera configuration and orientation (e.g., changes of the angle-of-view). Consequently, its application to real life traffic monitoring system is limited.

In this paper, we discuss a multi-camera tracking system for an intersection or a small parking. We assume that the monitored area has a finite number of entrances and exits. Moreover, the cameras can be deployed so that all cars entering the area are detected and fields of views (FoVs) of neighbouring cameras overlap partially. This ensures that when a vehicle leaves one camera’s FoV it is already in the FoV of its neighbour. In such deployment, when a vehicle is processed by a camera, the information about its detection, classification or tracking can be transferred to neighbouring camera. This may simplify image processing operations on the neighbouring cameras and enable multi-camera tracking. Information about the vehicles can be transferred in different ways but since it is needed locally (e.g., tracking) the use of local communication is preferred. Allowing device-to-device communication gives the ability to quickly exchange information about the vehicles and removes the necessity to include central decision unit in the system.

To address the requirements of this use-case we propose a Neuroflow system, which has two unique features:

- multi-camera vehicle tracking without number plate processing, implemented directly in the camera embedded system,
- local communication mechanism between cameras which allows to hand-over information about the vehicles.

These features allow building a distributed vehicle tracking system which follows the Edge Computing paradigm–all decisions are taken locally by cameras and only the final tracking of a vehicle is stored in the central system. Reduced complexity of the proposed image processing algorithms allows building a system based on single board computers which are relatively low-cost and power-efficient. Communication mechanism between the cameras is based on the ISM-band wireless communication, with added reliable network protocol to provide expected Quality of Service metrics. The system can be used for long-term monitoring as well as for temporary deployments (which are installed only for a limited time and then moved to another location). Moreover, the lack of ANPR processing guarantees privacy, which is an important element of traffic monitoring systems. This is a desired feature as in some countries the use of traffic monitoring systems based on ANPR is prohibited due to privacy concerns [10].

The contribution of this paper is as follows:

- implementation of single camera tracking algorithms (tracking by detection) that can be run in the camera embedded system (edge processing),
- multiple camera tracking that does not require central processing,
- vehicle tracking approach that is not based on the number plate recognition and can efficiently operate with low resolution images,
- the use of low-power ISM radio for local communication between the cameras in order to implement multi-camera tracking.

The next section compares contribution of this paper with state-of-the-art and summarizes the most important advancements of the presented method.

## 2. Related Work

Many different techniques are used for traffic control and vehicle detection, but vision-based ones are the most flexible [3,4,5,11,12,13,14]. For example, in Reference [13] authors show that different sensors can be used to monitor free parking spaces in open-access city-wide street parking scenario. Although magnetometers, inductive loops, infrared or ultrasonic sensors could be used in closed parking lots, their use outdoors is limited and economically inefficient, as the monitored places are dispersed over a wide area. Vision based systems are universally applicable to both indoor and outdoor scenarios, with broad range of use cases, not limited only to simple space-occupancy problems, but offering simultaneously vehicle detection, classification and tracking as well as enabling traffic law enforcement [5]. The installation is also simpler and cheaper, as the road surface is not affected. Vision based approaches to tracking can be categorized with respect to the number of cameras used in tracking (single/multi camera tracking), detection and tracking algorithms (e.g., type of convolutional neural network used for detection), and the general architecture of the system (the use of centralized or edge processing).

Multi-target single-camera (MTSC) tracking is the most often addressed, but applicable only to cases when the whole area of interest (e.g., intersection) can be covered by a single camera. This approach was followed by Seong et al. [15] who used a high resolution CCTV camera mounted on a pole to monitor the whole intersection. The captured images were transmitted to the processing unit and processed in real time. The proposed solution aimed at detecting positions and reconstructing the trajectories of all the vehicles in the intersection. The root mean square error (RMSE) calculated by based on the differences between the determined and the real ground-truth position was equal to 16.51 m.

Similar approach was used by Wang et al. [16] but the high resolution camera was mounted on an unmanned aerial vehicle (UAV) and video images were processed off-line. The proposed tracking-by-detection approach performs efficiently at 11 frames per second. The average F${}_{1}$ score for detection equals 92.1% and multi-object tracking accuracy (MOTA) equals 81.3%. Despite good results the proposed approach can only operate for a limited time, thus it is not suitable for continuous tracking. Moreover, the resulting system was sensitive to vehicle colour, for example, black vehicles were difficult to distinguish from asphalt and shadows.

Barthélemy et al. [17] presented a system that uses edge processing to implement single camera tracking-by-detection and object classification. The solution uses CCTV cameras that operate independently and uses low-power LoRa radios to communicate the results of the tracking to the central unit. Unfortunately, the performance of the system (both in terms of detection accuracy and the number of processed frames per seconds) is strongly affected by the number of objects in the video stream. For a large number of objects, both the number of frames processed per second and the accuracy of detection deteriorated.

Multi-target multi-camera (MTMC) tracking is more universal but also more challenging. In MTMC systems vehicles need to be tracked within the FoV of each camera and also between two or more cameras. Existing solutions to the second task either match a vehicle between the cameras using the number plates, re-identification (ReID) and feature matching, or cameras with overlapping FoVs.

CityFlow, presented in Reference [8], is a city-scale traffic camera dataset, consisting of videos from 40 cameras across 10 intersections. The longest distance between two cameras in this set is 2.5 km. The paper presents various off-line approaches to vehicle tracking within a single camera (MTSC) and vehicle ReID between different cameras. For the best set of algorithms (MTSC + ReID) the resulting performance (F${}_{1}$ score) is below 50%. According to the authors this is due to inefficiencies of both algorithms. The two challenges in the ReID are large intra-class and small inter-class variability, that is, the differences in shape of the same vehicle under different angles are large, while shapes of vehicles from different manufacturers are similar.

In the article by Jin [18] the same dataset of videos is used, but vehicles are tracked across a single intersection using multiple cameras. In this scenario the accuracy of ReID is 81.59% but the overall accuracy of the MTMC tracking is not provided. The article also reports high complexity of the ReID calculation that caused the single tracking to take approximately 2 s. This significantly narrows practical applications of this approach.

Nowadays, most of the MTSC tracking approaches use a convolution neural network for vehicle detection in a video frame [19], and dedicated algorithms (e.g., based on Kalman or particle filters [12,20]) for analysing vehicle movements based on multiple detections on consecutive frames. For object detection SSD and YOLO networks are often used as they are fast and accurate. However, they are also complex and computationally demanding [21]. Consequently, if detection needs to be conducted in real-time, the algorithms require high performance computers equipped with powerful GPUs and large amount of memory [15,16,18,22,23].

For example, detection approach presented in Reference [16] was using a fine-tuned YOLOv3 detector and was run on a computer equipped with 6-core, 12 threads CPU, NVIDIA GeForce GTX 1080 Ti and Intel UHD Graphics 630 GPU. YOLOv2 and central processing unit were also used in Reference [15], but the article does not provide information on the system components.

On the contrary, the article by Bottino et al. [24] presents a hybrid solution where detection uses an embedded system (CuBox-i4 Pro) and tracking is run on the PC workstation. However, their solution uses a different detection approach based on the segmentation and background subtraction in which the accuracy strongly depends on the environmental conditions and the type of the intersection (e.g., the number of lanes).

Edge processing for single camera detection and tracking was recently demonstrated by Barthélemy et al. [17]. They have used CCTV cameras equipped with Jetson TX2 single board computer that were running YOLOv3 detector and SORT tracking. However, the accuracy and performance of the system was strongly affected by the traffic load. In Reference [25] various detection and tracking approaches (including various versions of YOLO, SSD and SORT) were evaluated on several platforms. Presented results show that even a tiny variants of YOLOv2 and YOLOv3 detectors cannot achieve performance above 16 frames per second when run on Jetson TX2 module. The performance is even lower for standard YOLOv2/YOLOv3 and is not enough for practical applications.

Existing approaches to multi-camera tracking use convolutional neural networks both for tracking within the FoV of a single camera, and ReID and inter-camera tracking. For example, the system presented in Reference [23] (which achieved 70% correctly identified detections and performed the best during AI Challenge 2018 [7]) used ReID based on deep-learning and TrackletNEt Tracker that is based on convolutional neural network.

Majority of the efficient solutions which support MTSC and MTMC tracking (e.g., References [15,16,22,23]) use high performance central computer systems to process and analyse the captured videos. The algorithms (including SSD and YOLO detectors) are computationally and memory demanding and implementing them efficiently in constrained devices is a challenge (ensuring that sufficient number of frames per second is processed, as required for tracking). The straightforward implementation of inter-camera tracking in MTMC systems also requires central units to collect vehicle related information from multiple cameras, and run complex ReID algorithms.

So far, only some of the detection and tracking tasks were successfully deployed in the embedded systems (e.g., References [17,24,25]). Unfortunately, these either do not use convolutional neural networks [24] or do not perform efficiently enough to be used for reliable and long term tracking [17,25]. No practical edge processing solution was proposed so far for multi-camera vehicle tracking.

Table 1 compares the state of the art approaches to vehicle tracking using single and multiple cameras.

In contrast to previous results, our approach is based on edge processing where detection and tracking algorithms are fit into the low-power embedded system of the camera. It was possible thanks to the design of a dedicated detection algorithm that uses proprietary, hourglass-like convolutional neural network, and a system of cameras with overlapping FoVs. This set of features gives us the opportunity to create a multi-camera tracking system with edge processing.

## 3. System Architecture

### 3.1. Purpose of the System

The purpose of the designed system is to provide a low-power multi-camera traffic measurement tool that can be used for short and long-term traffic monitoring in various scenarios. The main applications of the designed system are as follows:

- parking monitoring and management—the system can provide information about parking spots occupancy, and this information can be used in further applications such as billing, route suggestions for drivers, traffic organization and so forth.
- intersections monitoring—for traffic surveys or day-to-day traffic management. In simple systems, usually only the vehicle queues are monitored, counting how many vehicles approach (or stop) on each incoming lane. This information can be used to increase the lengths of green cycles for the roads with highest traffic demand. However, a much more interesting measure are flow diagrams of vehicles, which show what are the most used relations that vehicles take (i.e., the counts of vehicles for each entry-exit pair). To take such a measurement, vehicle tracking across the whole intersection is necessary.

Furthermore, if the required measurements are a part of a short-term traffic survey, the designed system should be easy to install and configure, and operate efficiently on batteries for the requested time (usually 24–48 h). This simplifies the deployment as the process of getting proper permissions for AC-power supply is often difficult and time-consuming. On the other hand, energy-efficiency of a system is an important feature, also in long-term evaluation.

In all the scenarios, vehicle tracking is one of the key features of the system, to provide flow-related data, detect anomalies (e.g., vehicle stopped in unusual place) and identify vehicles (e.g., in order to monitor how long a parking spot has been used by a vehicle). This can be used for information/planning, as well as parking space retention enforcement.

### 3.2. Architecture

**Figure 1:**
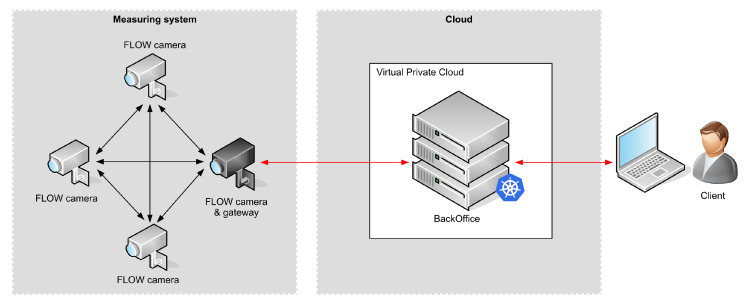
High level architecture of the vehicle tracking system. Black and red arrows denote local and global communication respectively.

The vehicle tracking system (Figure 1) is composed of a measuring system and a central server that collects information from the measuring system and provides it to the clients. Single server can communicate with a number of independent measuring systems deployed in different areas.

Each measuring system can be composed of up to 16 FLOW cameras. The cameras are responsible for image acquisition, vehicle detection, and tracking. Cameras are based on Jetson Nano platform which is a power efficient single board computer capable of running artificial intelligence algorithms at the edge in real time. Jetson Nano is the simplest in Jetson Family, equipped with Quad-Core ARM Cortex-A57 MPCore processor, 128-core NVIDIA GPU and 4 GB of memory capable of executing 472 GFLOPs. Cameras are equipped with a GPS receiver that provides them with location and accurate time synchronization that is used for time-stamping of vehicle detections. In this setup, we were able to run the system for around 24 h, when powered from a 21 Ah battery. The actual runtime depends on system workload, that is, the number of vehicles that need to be detected and traced.

Each camera can use two types of communication: local and global. Local communication is designed to be used for bidirectional communication between the cameras in order to exchange setup information, statuses and, most importantly—information about vehicles being tracked. The communication is based on a proprietary communication protocol utilizing 868 MHz frequency band [26]. It was designed specifically to meet the requirements of the vehicle tracking system, especially low latency. Information about detected vehicles allows a set of cameras to collect information about the route that every vehicle takes through the area covered by the measurement system. The camera that spots a vehicle leaving its FoV broadcasts the information about the vehicle to all cameras in the system. The camera that picks up a vehicle entering its FoV, correlates the information received over the radio with its own detection results and continues tracking the object. This procedure continues until the vehicle leaves the monitored area. The last camera that detects a vehicle leaving its FoV sends this information to the gateway camera. The gateway camera forwards tracking information to the external server.

The global communication is available in gateway cameras to enable communication with the Internet and the server. It is primarily used for collecting information about tracked vehicles but also for remote management and monitoring of the measuring systems. In the experimental setups all cameras were equipped with global communication (using LTE ) in order to collect raw images for offline verification of the detection and tracking algorithms.

The central server is responsible for storage of the information from the cameras and provides data to the clients. In the experimental setup it was also responsible for reconstruction of vehicle trajectory across the measured area based on the information received from the cameras.

The architecture using both local and global communication was designed to meet specific requirements of the tracking system. Processing video images in the cameras offloads the communication interface, as high resolution video streams do not need to be transmitted. The proposed edge processing also improves the overall privacy, as images may never leave the camera and can be removed as soon as anonymous tracking information is extracted and the vehicle leaves the camera’s FoV. Extracted tracking information contains information about random vehicle id, timestamp, tracking event type, vehicle path and parameter that estimates the credibility of tracking. This information is below 30 bytes that need to be transmitted between the cameras of the measurement system. Because there is not much data to transmit, the local communication does not require high data throughputs. Instead, it should ensure reliable and low latency communication, as this reduces processing delay in the chain of cameras that track the vehicle.

### 3.3. Vehicle Detection and Tracking

**Figure 2:**
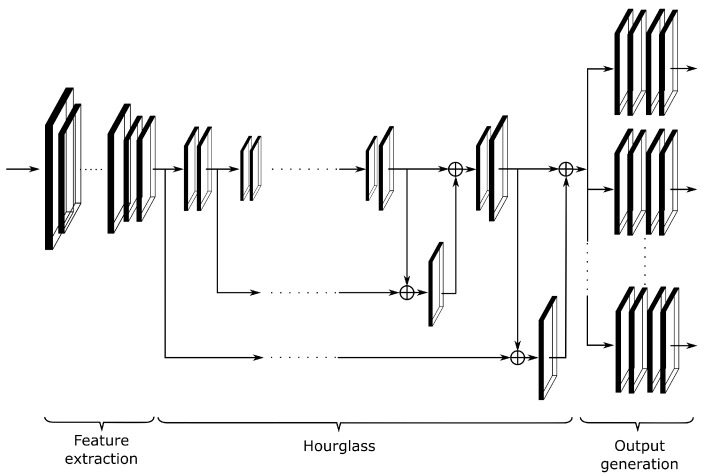
General structure of the neural network used for vehicle detection.

The cameras use a dedicated deep learning algorithm to process video snapshots and detect vehicles. The detection algorithm is based on the convolutional neural network that processes each snapshot directly on board of the camera. Due to constrained resources and performance of the single board computers that were used, we decided not to use SSD and YOLO networks, but design a proprietary neural network (Figure 2). The network is adjusted to the application and downsized. Compared to YOLO and SSD it has less coefficients by the order of magnitude, requires significantly less memory, and the number of classes is reduced and adjusted to application characteristics. While the network cannot detect people, trams, or bicycles, it can, for example, distinguish trucks from trucks with trailer. The network classifies vehicles into official vehicle classes (such as motorcycle, passenger car, bus, semi-truck, truck, truck with trailer, etc.). To further improve the performance, the network is trained using floating point arithmetic, but for the detection, the coefficients are quantized and reduced to 8-bit fixed-point representation. The resulting network has almost 7,600,000 coefficients that require almost 16 MB of memory. A single detection on a Jetson Nano single board computer takes approximately 100 ms. Because training of the network requires large amount of data and processing, it is done in the external system using GPU cards. The resulting network is then uploaded to the cameras.

**Figure 3:**
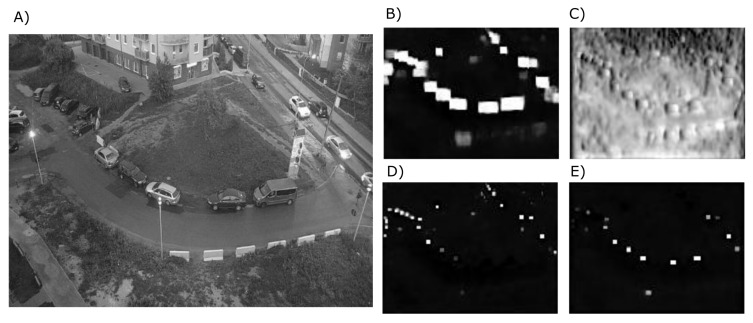
Example of detection input (**A**), and the output: heatmap (**B**), bounding box offsets (**C**), detection at scale 0 (**D**) and scale 1 (**E**).

The network takes 512 × 384 pixels monochrome images as inputs and processes them in three main steps—first, it extracts the features, then processes the features in hourglass-like neural network, then finally generates the outputs. The output (Figure 3) is composed of a detected class, heatmap, bounding boxes and offsets generated in four different scales, each of 128 × 96 pixels.

The systems use tracking by detection for both single-camera tracking and multiple-camera tracking. For single-camera tracking, vehicles detected on each video frame are assigned a bounding box, location, and a timestamp. Detection results from subsequent snapshots (frames) are fed to the tracking algorithm. The algorithm links the vehicles detected at the snapshots, taking into account locations of the bounding boxes, timestamps and the physics of the vehicle movement (e.g., speed). As a result, each camera assigns each vehicle a unique identifier and a timestamped history of vehicle locations within the camera’s field of view. Multi-camera tracking benefits from the timestamps and the fact that the fields of view of the cameras overlap. When a vehicle leaves the camera’s field of view, the camera sends the tracking information to its neighbours. Based on timestamps, the camera that picks up tracking is able to decide which vehicle (from the previous camera) it is tracking. Because unique identifiers are generated randomly and are not related to the vehicle identity (e.g., the number plate or, VIN) the multi-camera tracking does not expose privacy. Inter-camera tracking can be either run directly on the cameras or on the central server. For the first scenario the picking camera correlates information received over the radio with the results of its own detection. Because the FoVs partially overlap, the picking camera detects the vehicle a moment before the vehicle leaves the previous camera FoV and as it broadcasts the tracking information. This pattern continues and successive cameras accumulate information about the tracked vehicles. Operation of the system is similar in the centralized scenario except that all the cameras send their individual tracking information to the server. The server performs inter-camera tracking based on the timestamps and the information about the parameters of the measurement system, in particular—the FoVs of all the cameras in the system.

### 3.4. Local Low-Power Radio Communication

Cameras communicate with each other using 868 MHz ISM band. The 2.4 GHz was briefly tested at the beginning of the project but the limited communication range and high susceptibility to traffic-generated radio noise proved them unusable for the required application. On the other hand, in 868 MHz band we were able to achieve communication range between cameras in the order of several hundred meters as well as packet reception rates close to 100%. The drawback of 868 MHz communication is the available bandwidth, with the default set to 50 kbps and the maximum being 200 kbps (but at the cost of data reliability and communication range).

All cameras in the measurement system form a self-organized ad-hoc network. We use both CSMA (Carrier Sense Multiple Access) and TDMA (Time Division Multiple Access) techniques to minimize the effects of packet collisions and the need of packet retransmissions. A protocol has been designed to allow nodes (cameras) to join the network and allocate timeslots for all the devices to maximize the bandwidth usage. It allows forming ad-hoc network with only two nodes or whenever more nodes join the network. In the network one of the nodes becomes responsible for sending beacons which synchronize the network operation, but other ways of synchronization are possible. For example, the nodes can use lightweight time synchronization protocols (such as References [27,28]), or external synchronization signals (e.g., from GPS receivers) if in-network synchronization is troublesome or inefficient due to the size of the network.

Messages that are sent between cameras may be split into two categories—vehicle data and maintenance/control messages. Data about recognized vehicles from one camera may be needed in more than one device along the way, so to allow efficient communication, we use broadcast messages. To avoid unnecessary overhead, these do not require immediate acknowledgements, but they are marked with sequence numbers, so when a receiving system detects some missing frames, it may request their retransmission. On the other hand, validity of vehicle data is time-limited. Currently we assume that after 30 s the vehicle data from one camera becomes useless for other cameras in the system, so it is purged and cannot be retransmitted even if requested. This should not normally happen, but if the queues of unsent messages grow due to temporary communication problems, it is better to drop messages that are no longer valid, rather than clog the system for extended period of time.

Control messages, on the other hand, are handled with high priority—they may contain reconfiguration commands, as well as retransmission requests, or policy adjustments that affect the radio communication. There is a separate queue for these messages, and each sent message requires an acknowledgement. Statistics is gathered for all packet transmissions, acknowledgements and retransmissions, which can be used to change communication parameters, such as transmit power or radio channel used.

## 4. Evaluation in Pilot Locations

**Figure 4:**
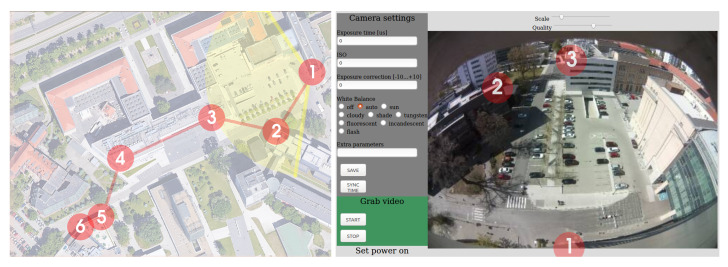
Deployment of the test system at university campus—camera locations and camera #1 FoV.

**Figure 5:**
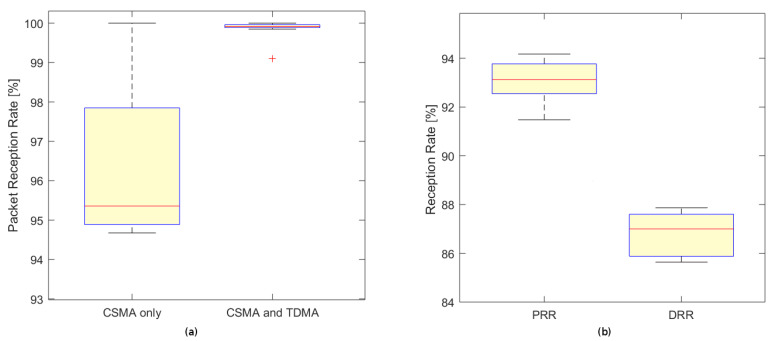
Packet reception rate (PRR) during normal operation (**a**) and PRR/DRR values under heavy traffic (**b**).

Several test locations have been used to test the system operation in real-world scenarios (Table 2). The first one was the university campus parking lot, where six cameras have been installed along one of the internal alleys (Figure 4). The parking lot entrance with a barrier is to the left of camera no. 6, and additional entry/exit barriers are in the area near camera no. 1. This was a test-bed installation primarily focused on evaluation of low-power radio communication (Figure 5) which required easy device access if manual reset/reprogramming was needed, as well as easy access to mains power supply. Due to that fact, we used cameras with narrow-angle lenses and cameras were placed on the rooftops of the surrounding buildings. Typically, wide-angle lenses are used and cameras are mounted at much lower heights–on traffic light poles or street lighting poles.

The second pilot installation was used for evaluation of vehicle detection. Evaluation was conducted on videos recorded as a reference during general traffic measurements (GTM). The GTM was conducted in early 2020 at several locations in Poland, on national roads. For the evaluation of vehicle detection, videos from 9 different locations have been used.

**Figure 6:**
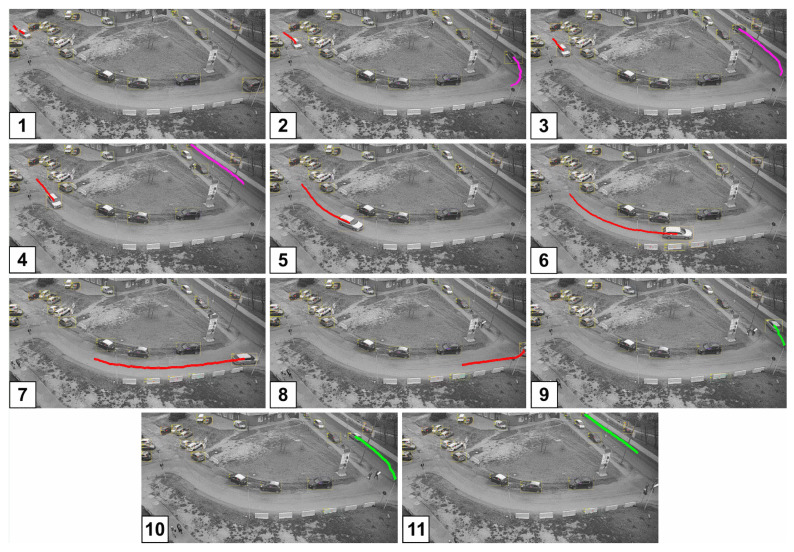
Example of single camera tracking. Images 1–11 present subsequent snapshots of the recording with two vehicles tracked. The first vehicle is visible on frames 1–4, the second one on all the snapshots. On snapshot 9 the vehicle is incorrectly identified as a new one.

**Figure 7:**
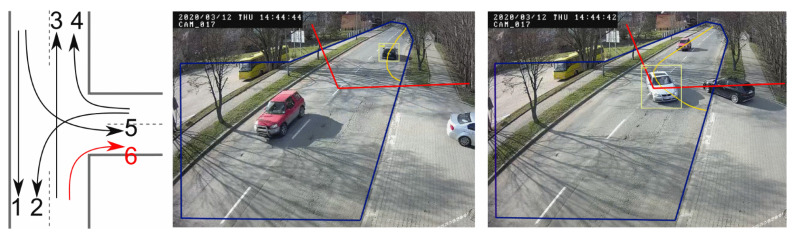
Possible trajectories of vehicles (trajectory 6 is not tracked) and examples of tracking for trajectories 4 and 5. Blue line marks the region of interest—objects outside are excluded from tracking. Red line is a line of detection—vehicles crossing this line are automatically assigned to trajectory 1 or 3, depending on the direction they move. For clarity, each of the vehicle tracked is presented on a separate snapshot.

The third pilot installation included two intersections—the first one was a residential area (Figure 6), and the second one—a suburban road with an intersection (Figure 7). First location was used for the development and evaluation of single camera detection and tracking. A camera was mounted on the 4th floor of the building and overlooked the whole area. The second location was used for evaluation of single camera tracking in real-life setup with camera located on a street lighting pole. In this location the system was detecting, classifying and tracking flows of the vehicles (i.e., deciding which direction vehicles came from and have gone to).

**Figure 8:**
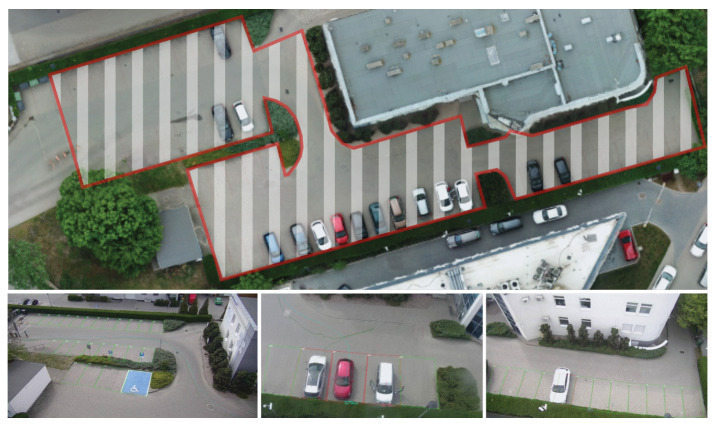
Simple multi-camera tracking system for a parking. The cameras track the vehicles from the barrier to the parking space.

The last location was the restricted entrance parking lot where the mulit-camera tracking was developed and tested. The parking (Figure 8) is equipped with single ANPR camera located at the entrance barrier and three FLOW cameras that perform tracking by detection. The FLOW cameras are located at the rooftop of the 4 storey building and cover the whole parking area.

### 4.1. Local Low-Power Communication

The low-power communication for FLOW cameras was tested in the measuring system deployed at the university and composed of 6 cameras. The system was deployed along internal, straight, 250 m long road located at the University campus (Figure 4).

The cameras were mounted at different heights on rooftops having no major obstacles in their line of sight. Due to relatively low traffic at the pilot site, the cameras simulated the traffic by generating vehicle tracking information at different intervals. This allowed us to simulate different traffic loads ranging from low up to extremely heavy traffic of 6 vehicles per second. Broader description of tested scenarios, results and discussion has been presented in Reference [26].

The main goal of the tests was to verify the operation of the system and its robustness. During the tests a number of parameters were registered and analysed, including data reception rate (DDR), packet reception rate (PRR), channel access efficiency (distribution of back-offs in CSMA procedure), duty cycling parameters (required for fair-use of the ISM bandwidth) and others.

With moderate vehicle (and radio) traffic the packet reception rate for all the sensors, when using TDMA and CSMA was close to 100%. When only CSMA was used the PRR varied between 95% and 98% (Figure 5a), but the retransmission mechanism allowed us to keep the DRR above 99%. When both TDMA and CSMA were used the DRR of 100% was obtained for all the cameras. Because DRR does not count control frames but only those that contain vehicle data sent between cameras it is a true metrics of system effectiveness.

For extremely heavy traffic (6 vehicles per second on all cameras) both the PRR and DRR dropped. While the change in PRR was moderate, the drop in DRR was significant (down to 86–88%—Figure 5b) primarily due to the strategy of dropping the vehicle tracking information that was considered out-of-date. This is the case in traffic monitoring as vehicles may leave the monitored area before the radio messages are successfully transmitted. This policy is specific to our application and preserves communication bandwidth for new vehicle-related information but lowers the overall DRR as a trade-off.

### 4.2. Vehicle Detection

The proposed vehicle detection approach was tested in various scenarios. This included parking areas, city and suburban roads, different speeds and traffic intensities as well as different camera settings including angles and distances of observation.

One of the test was conducted on national roads over the period of 16 h during a day and night time. The cameras were deployed in 9 different locations in order to count and classify vehicles. The video streams from all cameras were recorded for future processing and as a reference to evaluate the performance of the detection. Recordings were manually tagged, that is, over 55,700 vehicles that were recorded in the video were assigned a timestamp and a class information. The proposed vehicle detection approach was run on these recordings to assess its accuracy. Only 2020 vehicles were detected incorrectly which yields accuracy of detection above 96%. The missing detections were mostly due to extreme lighting conditions and vehicle speed (current implementation of the detection algorithm can process 11 frames per second, which is not enough for fast moving vehicles).

### 4.3. Single-Camera Tracking

Figure 6 presents snapshots of single camera tracking on two streets and part of an intersection. The camera is located on the roof of the 4 storey building and overlooks the area of approximately 2500 m^2^.

The camera tracks moving vehicles drawing colour trails for each vehicle. In the presented example there are two moving vehicles: first (vehicle A-magenta trace) on snapshots 1 through 4, and second (vehicle B, red and green trace) on all 11 snapshots. The vehicle B passes across the cameras FoV (snapshots 1–7) to the edge of the FoV (snapshot 8 and 9). Because the intersection is only partially visible, and vehicle B almost disappears from the FoV (snapshot 8) the tracking algorithm fails. As a result, although the vehicle B is detected and tracked on subsequent snapshots (9–11) the system considers it as a different vehicle.

Single camera tracking was also verified on a suburban road with T-shape intersection (Figure 7). The camera is mounted on a street lighting pole and traces moving vehicles. This is shown by drawing yellow trails for each vehicle correctly tracked (trails mark locations of estimated center of mass of the vehicle). The camera traces objects detected within the region of interest (ROI), decides each vehicle trajectory (from where and where to the vehicles goes), and automatically counts vehicles intersecting the detection line. Because the main purpose of the camera deployment was to capture and count traffic on the main road, its location and orientation does not allow to detect and count vehicles for all possible trajectories. Out of 6 trajectories that a vehicle can take on this intersection, one (incoming from the bottom, turning to the right) is not fully covered by the camera’s FoV and the ROI. Consequently vehicles turning right are not traced. Additionally, vehicles that do not intersect the detection line are traced but not counted automatically. Therefore, for our evaluation, vehicles going along trajectory 2 and some of the vehicles along trajectory 4 had to be counted manually (despite being tracked automatically). For the purpose of tracking validation, a valid trace has to meet three criteria:

- center of mass of the detected vehicle falls within the ROI,
- trace is at least 1 s long,
- trace is inline with trajectories defined for the location.

The last criteria can be defined in a number of ways. For example, how close the detected trace resembles the ideal track defined for each possible trajectory. For the sake of simplicity we decided that a trace is correct, if it correctly points to the direction from where the vehicle came and were it went to. Traces generated by the system are compared with human assessments. The resulting tracing accuracy is a ratio of those two values. The evaluation was conducted on a 6-hour long video recorded during the daytime. On this recording the human operator tagged a total of 932 vehicles. Out of this number, 564 vehicles travelled the main road (trajectories 1 and 3) and 368 made turns (trajectories 2, 4, 5 and 6), including 61, that were incoming from the bottom and turning right (trajectory 6). Tracking accuracy on trajectories 1–5 varied between 97% and 99.7% with an average accuracy exceeding 98%. Table 3 presents results of the validation for each trajectory. The table shows also one vehicle travelling trajectory 6. It turned out the vehicle was driving slowly enough, so the trace took longer than 1 s within the defined ROI.

When single camera tracking by detection is implemented in the embedded system, the tracking capabilities are limited by the performance of the system—the number of detections per second the camera can calculate, and the number of detected vehicles to track. The proposed convolutional neural network can run detection on 11 frames per second regardless of the number of vehicles in the FoV. Tracking time, however, depends on the number of objects being tracked - for between 15 to 20 vehicles it takes approximately 150 ms. As a result processing a single frame takes approximately 200–250 ms This limits the single camera tracking frequency down to 4–5 frames per second. The proposed tracking by detection approach provides correct tracking if IoU (Intersection over Union) for the boxes representing moving objects on the subsequent frames is at least 0.3. For a typical car this translates to a shift of slightly more than 2.6 m between frames and an approximate speed of 11 m/s ≈ 40 km/h. The achieved processing performance is sufficient for parking applications, as well as internal roads and intersections, where vehicles move slowly. Unfortunately, this is too low for tracking in general traffic conditions.

### 4.4. Multi-Camera Tracking

For parking lot scenario, a small parking was monitored with three FLOW cameras (Figure 8) located at the rooftop of the neighbouring building. Additional ANPR camera was located at the entrance barrier to verify if the vehicle can enter the parking. The cameras tracked vehicles through the area with the goal of measuring parking time of each vehicle in the monitored area.

Although the FLOW cameras cannot recognize the number plates of the cars, the system can tell where each car is parked—that is, assign the number plates read at the barrier to the location where the vehicle finally parked. This is possible because the number plates are captured at the barrier and timestamped. Simultaneously, FLOW cameras detect the vehicle, assign them a unique identification number, and track the vehicle through the parking area. Based on the identification number and timestamps each trace can be correlated with the number plate and used for the estimation of parking duration, when vehicle stops in the parking spot.

Multi-camera tracking has not yet gone long-term validation and was tested on small number of vehicles, also because the parking area monitored is only occupied during daytime (working hours). This prevents thorough validation in different environmental conditions (e.g., at night). However, since the multi-camera tracking requires each camera in the chain to track the vehicle correctly, thus the expected accuracy of multi-camera tracking can be estimated as a product of single camera tracking accuracies. Based on the results of single camera tracking, we can assume single-camera tracing accuracy of at least 97%. For 3 cameras in the multi-camera system this yields accuracy of correct tracking to be at least 91%.

## 5. Conclusions

This paper presents the concept of traffic monitoring system based on deep neural networks and edge processing. The system consists of several components, where each of the components should be treated as a crucial building block which allows to compose a final solution. The components of the system were verified in several real live scenarios proving high accuracy of vehicle detection and tracking in single camera scenario. Multiple camera tracking was also presented and proved but requires more in-depth evaluation for larger number of vehicles as well as various environment conditions. The proposed image processing approach, including detection and tracking, can be executed on low-power embedded devices in cameras. Presented solution is based on edge-computing paradigm—data is processed locally by cameras in order to perform single camera tracking. Moreover, we have proposed a dedicated low-power radio communication to exchange data between the cameras, in machine-to-machine manner. This ability is used to exchange vehicle tracking information between neighbouring cameras in order to implement multi-camera vehicle tracing. The energy usage of the system is low—camera embedded device consumes 20 W at maximum, and can run on 21 Ah battery pack for almost 24 h. This enables our devices to be powered from batteries and deployed in different locations where external power supply is not available or problematic.

Future work covers extended evaluation of the above components in a production environment, in different scenarios (e.g., large number of vehicles, day/night and weather conditions). Besides that, we plan to work towards increasing the efficiency of image processing algorithms. We also plan to use more powerful Jetson Xavier NX single board computer. Higher computational capabilities and deep learning accelerator will allow increasing the number of frames processed per second. This will give the opportunity to employ the system in higher-speed traffic and will broaden the range of deployments.

## Figures and Tables

**Table 1:** Comparison of different approaches to vehicle tracking.

Article	Single/Multiple	Intersection/City	Central/Edge	Detection	Long Term
	Camera	Level	Processing	Algorithm	Tracking
[15]	single	intersection	central	YOLOv2	yes
[16]	single	intersection	central	YOLOv3	no
[24]	single	intersection	partial edge	segmentation	yes
[22]	single	street	central	HOG, FasterR-CNN	yes
[17]	single	intersection/street	edge	YOLOv3	yes
[18]	multiple	intersection	central	CenterNet	yes
[23]	multiple	city	central	TrackletNet	yes
[8]	multiple	city	central	YOLOv3, SSD512	yes
our	multiple	intersection/parking	full edge	hourglass	yes
			possible	proprietary	

**Table 2:** Summary of the pilot installations.

#	Location	Number	Purpose of Evaluation
		of Cameras	
1	University parking lot	6	low-power communication
2	National roads, 9 locations	1 per location	vehicle detection
3	Intersection, 2 locations	1 per location	vehicle detection, single camera tracking
4	Parking lot	3	multi camera tracking

**Table 3:** Results of a single camera tracking evaluation for each possible trajectory (cf. Figure 7) including trajectory 6 which was not intended for tracking due to inadequate deployment of the camera.

Trajectory	Total Vehicles	Vehicles Correctly Tracked	Tracking Accuracy
1	299	298	99.7%
2	109	107	98.2%
3	265	264	99.6%
4	108	105	97.2%
5	90	89	98.9 %
6	91	1	1.6%
